# Nanomaterials as carriers to improve the photodynamic antibacterial therapy

**DOI:** 10.3389/fchem.2022.1044627

**Published:** 2022-11-25

**Authors:** Houhe Liu, Yuan Jiang, Zhen Wang, Linping Zhao, Qianqian Yin, Min Liu

**Affiliations:** ^1^ College of Agriculture and Forestry, Linyi University, Linyi, China; ^2^ Clinical Medical College and The First Affiliated Hospital of Chengdu Medical College, Chengdu, China; ^3^ The Fifth Affiliated Hospital of Guangzhou Medical University, Guangzhou, China; ^4^ Longhua Hospital Affiliated to Shanghai University of Traditional Chinese Medicine, Shanghai, China

**Keywords:** photodynamic antibacterial therapy, photosensitizer, nanomaterials, reactive oxygen species, non-antibiotic

## Abstract

The main treatment for bacterial infections is antibiotic therapy, but the emergence of bacterial resistance has severely limited the efficacy of antibiotics. Therefore, another effective means of treating bacterial infections is needed to alleviate the therapeutic pressure caused by antibiotic resistance. Photodynamic antibacterial therapy (PDAT) has gradually entered people’s field of vision as an infection treatment method that does not depend on antibiotics. PDAT induces photosensitizers (PS) to produce reactive oxygen species (ROS) under light irradiation, and kills bacteria by destroying biological macromolecules at bacterial infection sites. In recent years, researchers have found that some nanomaterials delivering PS can improve PDAT through targeted delivery or synergistic therapeutic effect. Therefore, in this article, we will review the recent applications of several nanomaterials in PDAT, including metal nanoclusters, metal-organic frameworks, and other organic/inorganic nanoparticles, and discuss the advantages and disadvantage of these nanomaterials as carriers for delivery PS to further advance the development of PDAT.

## Introduction

Bacterial infection is a great challenge to human health and a serious threat to public health security (Alzahrani et al., 2018; Wang et al., 2022). To date, chronic or acute diseases caused by pathogenic bacteria, such as wound infections, pneumonia, meningitis and sepsis, still threaten human life (Bleiziffer et al., 2017). In 1928, American bacteriologist Alexander Fleming discovered the world’s first antibiotic, penicillin (Vermassen et al., 2019; Kondo et al., 2022). Penicillin was mass-produced in 1940, ushering in the golden age of antibiotics (Zhou et al., 2022b). The discovery of antibiotics has opened up a revolutionary application in the field of medical science, showing good therapeutic effects on some incurable inflammatory diseases, saving the lives of countless patients (Cui et al., 2021; Kotwani et al., 2021). To this day, antibiotics are still used for the treatment of various bacterial infectious diseases. However, due to the widespread use of antibiotics and the rapid mutation of bacteria, the emergence of bacterial resistance has exposed the limitations of antibiotic application (Ma et al., 2018; Mil-Homens et al., 2021; Chang et al., 2022; Chawla et al., 2022). Routinely developed new antibacterial drugs, due to their single target, cannot alleviate the generation of bacterial resistance, and will lead to a decrease in the antibacterial efficiency of the drug over time (Xu and Yang, 2020). Light has been used in the treatment of disease for thousands of years, and there has been a lot of research into the treatment of disease (Ouyang et al., 2020). Photodynamic therapy (PDT) means that after the photosensitizer is excited by suitable wavelength light, it produces toxic substances such as singlet oxygen and free radicals, which act on the target cells and kill them (Odorici et al., 2021). PDT was first reported in 1900, when German scientist Oscar Raab found that acridine dyes activated by lightning could inactivate paramecium, while any single action of acridine dye and lightning could not kill paramecium (Mansoori et al., 2019; Solarte et al., 2022). At present, PDT has been approved by the United States, France, the Netherlands, Canada and other countries for the clinical treatment of tumor and non-tumor diseases within a certain range (Jin et al., 2020). In recent years, photodynamic antibacterial chemotherapy has been proposed as an alternative to PDT antibacterial therapy, and it has attracted much attention because of its obvious killing effect on multidrug-resistant bacteria (Begum et al., 2020; Mei et al., 2020). PDAT will not cause drug resistance problems due to factors such as a single drug, the concentration of PS and insufficient exposure time.

However, photodynamic antibacterial therapy is not perfect, and the hydrophobicity and lack of targeting of PS are the biggest challenges for photodynamic antibacterial therapy. A large number of studies have shown that the use of nanotechnology to deliver PS is an effective way to solve the dilemma faced by photodynamic antibacterial and can enhance the effect of photodynamic antibacterial (Miao et al., 2022; Xu et al., 2022). Therefore, in this article, the application of nanotechnology in photodynamic antibacterial therapy will be reviewed, including delivery principles, nanostructures, and future prospects for photodynamic antibacterial therapy. The information of all nanomaterials is shown in Table 1.

### Photodynamic antibacterial therapy

**FIGURE 1:**
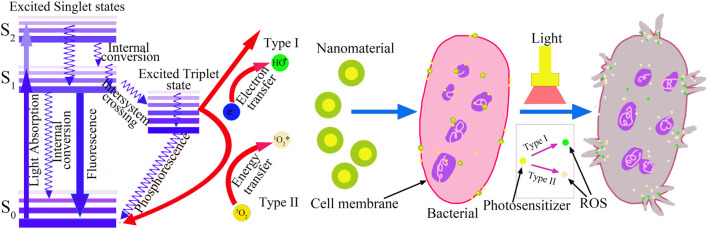
Photochemical mechanisms and biological mechanism of PDAT.

There are two main types of photochemical mechanisms of PDAT (Figure 1), including type I reactions and type II reactions (Gupta et al., 2021; Lin et al., 2021; George et al., 2022). Among them, the type I reaction means that after the photosensitizer enters the bacteria, under the irradiation of light of a certain wavelength, the photosensitizer that absorbs photon energy transitions from the ground state S0 to the excited state S1, in which part of the energy is radiated in the form of fluorescent quantum, and the remaining energy will be photosensitive (Winifred Nompumelelo Simelane and Abrahamse, 2021; Donzello et al., 2022; Yu et al., 2022). The agent molecules lead to the excited state T1, which is also called intersystem crossing. The excited state T1 is the appropriate form of the photosensitizer in the PDT process. In the excited triplet state T1, photosensitizers can transfer ambient energy to biomolecules, which further react with oxygen to form lipid peroxides, which disrupt the structural integrity of bacterial cell membranes and increase the ionic permeability of bacterial cell membranes (Mei et al., 2020; Odorici et al., 2021). The way the PS in the excited state T1 transfers energy directly to O_2_ is type II reaction. The O_2_ excited in this way is the so-called singlet oxygen (^1^O_2_), which is characterized by an extremely strong oxidizing power and is capable of causing oxidative damage to bacteria. Most organic compounds are in the ground state, while oxygen molecules are characterized by their triplet state (which is the basis) that can be excited to a singlet state. In type II reactions, the excited photosensitizer can react with triplet oxygen molecules. In the process of photodynamic therapy, the above two mechanisms usually occur at the same time. However, as oxygen is depleted, type I reactions begin to dominate because they are less dependent on oxygen (Zhang et al., 2019; Kwon et al., 2021; Liu et al., 2021).

**TABLE 1:** Summary of nanomaterials for PDAT.

Types of nanomaterials	Name of nanomaterials	Photosensitizer	Application	References
Hollow mesoporous silica nanoparticles	UHSN@CS	Ce6	*S. aureus* (*S. aureus* infected dermal wounds)	Yan et al. (2021a)
Upconverting nanoparticles	UCNPs	Indocyanine green	*S. aureus* and *E. coli* (*S. aureus* infected abscess)	Zhou et al. (2022a)
Polymer nanoparticles	DMCPNs	Poly [2-methoxy-5-(2-ethylhexyl)oxy)-p-styrene]	Amp” *E. coli* (Not specified)	Zhang et al. (2020)
Gold nanoparticles	AuPNs	Indocyanine green	*S. aureus* (Not specified)	Wu et al. (2021)
Polydopamine nanocomposite	PDA-Cur nanocomposite	Curcumin	*S. aureus* and *E. coli* (Not specified)	Su et al. (2021)
Carbon dots nanoparticles	CDs/Cur	Curcumin	*S. aureus* and *E. coli* (Not specified)	Yan et al. (2021b)
Graphene oxide nanomaterial	NGO	Graphene oxide	*S. aureus* and *E. coli* (Not specified)	Romero et al. (2020)
Aggregation-induced fluorescence nanomaterial	TPA-18	TPA-18	*S. aureus* and *E. coli* (Not specified)	Yang et al. (2020)
	AIE-NFs, NFs-K18	AIE-NFs, NFs-K18	MRSA (MRSA wound infection)	Guo et al. (2022)
Metal organic frameworks nanomaterial	PCN-224 (Zr/Ti)	PCN-224 (Zr/Ti)	*S. aureus*, *E. coli*, MDR *E. coli*, MDR A, MRSA and MRSE (MRSA wound infection and MDR *E. coli* wound infection)	Chen et al. (2020)
Silver nanomaterial	AgNCs/RB	RB	*Streptococcus* mutans, Porphyromonas gingivalis and Aggregobacter actinomycetes (Not specified)	Shitomi et al. (2020)
Nano-graphene quantum dots	GQDs@hMSN(EM)	GQDs	*S. aureus* and *E. coli* (*S. aureus* wound infection and *E. coli* wound infection)	Wang et al. (2020)

### Hollow mesoporous silica nanoparticles

With the rapid development of nanotechnology, various nanomaterials have been widely developed and applied to improve drug properties. Hollow mesoporous silica nanoparticles has been widely used in the field of drug carrier and antibacterial infection due to their large cavity, regular pore structure, high specific surface area, stable chemical and mechanical strength, easy surface functional modification, good water dispersibility and excellent biocompatibility (Zeng et al., 2021; Wu et al., 2022). In addition, the difficult healing of wounds caused by biofilms requires more antimicrobial biologics to be developed (Ouyang et al., 2022a). Yan et al. (2021a) used the hard template method to prepare a series of hollow silica nanoparticles with different thicknesses (∼30 nm, ∼20 nm, ∼10 nm) and then modified with chitosan to obtain UHSN@CS. UHSN@CS exhibited high loading efficiency (80.6%, pH = 6.0) and controllable Chlorin e6 (Ce6) release by pH-responsive. Furthermore, UHSN@CS could enhance the ROS production of PS and effectively adhere to *Staphylococcus aureus* (*S. aureus*), thereby greatly enhancing the antibacterial properties against bacteria. UHSN@CS-Ce6 can wash the mature *S. aureus* biofilm and reduce the biomass by 81%, and the treatment effect under laser irradiation is better than that of Ce6 (59.2%). *In vivo* experimental results confirmed that compared with Ce6 (50 μL, 100 μg ml^−1^) treatment group, UHSN@CS-Ce6 (50 μL, 100 μg ml^−1^) treatment group could promote the regeneration of infected wound more effectively under the same light irradiation (10 min, 660 nm, 5 mW/cm^2^). These results indicate that mesoporous silica nanoparticles have a positive effect on PDAT.

### Upconverting nanoparticles

There is a hypoxic environment in deep inflammatory tissues, and with the increase of depth, the effect of PDAT is limited by the limitation of light penetration ability, which is also the biggest limitation faced by PDAT (Yuan et al., 2022). On this basis, Zhou (Zhou et al., 2022a) designed a near-infrared driven nanoplatform composed of tin disulfide (SnS_2_) nanosheets (POS NSs), UCNPs and ICG molecules (POS-UCNPs/ICG) by simultaneously generating O_2_ and CO are used in antibacterial therapy and have been proposed to reduce inflammatory responses in deep infections. Using a single 808 nm light, ICG molecules can achieve PDAT, while POS NSs generate O/CO through upconverting photoexcitation. In the process of PDAT, O_2_ can enhance PDAT, and CO can control the inflammatory response through the PI3K/NF-KB pathway. The POS-UCNPs/ICG group had the highest percentage of healing area in the mouse abscess model, reaching 91.55%. The poss-ucnps/ICG complex significantly accelerated recovery in animal abscess models by enhancing PDAT and anti-inflammatory synergistic therapy. This near-infrared light-responsive nanoplatform has optimized antibacterial ability and immunomodulatory functions, and is expected to be used for clinical treatment of bacterial infections.

### Polymer nanoparticles

The infection sites caused by many bacteria are accompanied by hypoxia. At the same time, the tissue penetration ability of white light is weak, which affects the efficiency of PDAT (Hajdamowicz et al., 2019). Zhang et al. (2020) designed and explored the efficient killing of ampicillin-resistant *Escherichia coli* (Amp” *E. coli*) by combining photothermal therapy (PTT) and PDT with bimodal antimicrobial conjugated polymer nanoparticles (DMCPNs). In the presence of (styrene-maleic anhydride), the photothermal agent poly (diketopyrrole-thiophenethiophene) (PDPPTT) and the photosensitizer poly [2-methoxy-5-(2-ethylhexyl)oxy)-p-styrene] (MEHPPV) nanoparticles were prepared by co-precipitation method. Therefore, DMPCNs have both a photothermal effect and the ability to stimulate the surrounding oxygen to generate ROS and destroy drug-resistant bacteria under illumination. The inhibition rate of DMCPNs at a concentration of 9.6 × 10^−4^ mM against Amp” *E. coli* was up to 93%, which was higher than the inhibition rate of PTT or PDT alone. Bimodal nanoparticles offer the potential for clinical treatment of pathogenic infections caused by drug-resistant microorganisms.

### Gold nanoparticles

With the development of PDT and PTT, the combined application of PDT and PTT provides a very potential therapeutic scheme for the development of antibacterial therapy (Meng et al., 2022). Therefore, it is very necessary to design an efficient joint scheme. Wu et al. (2021) used porous gold nanoparticles (AuPNs) as carriers to deliver the photosensitizer indocyanine green (ICG) to prepare self-assembled nanoparticles with near-infrared (NIR) light control, PTT and PDT dual effects. AuPNs can not only transform near-infrared light to thermal energy, but also give a porous structure with high loading efficiency for delivering ICG molecules. Through the electrostatic effect and hydrophobic interactions between the surface of AuPNs and ICG, ICG molecules were adsorbed on the surface of AuPNs, and the aggregation state of ICG observably enhanced the generation capacity of ROS. Through the combination of PDT and PTT, the nanoplatform showed a strong antibacterial effect against the Gram-positive pathogen *Staphylococcus aureus* under laser irradiation.

### Polydopamine nanocomposite

Polydopamine is a polymer of dopamine, which is widely used in the fields of chemistry, biology, medicine, materials science and applied science and engineering. Su et al. (2021) prepared a mixture of polydopamine and curcumin (PDA-Cur nanocomposite) by oxidative autopolymerization of dopamine hydrochloride which has good bacteriostatic effect against Gram-positive and Gram-negative bacteria. PDA-Cur nanocomposite exhibits great antibacterial activity against *S. aureus* and *E. coli*. PDA-Cur with 405 nm light can achieve 100% bactericidal rate for *Staphylococcus aureus* at low concentrations of 10 nM and 100% bactericidal rate for *Escherichia coli* at 1 nm. Under the simultaneous irradiation of 405 nm and 808 nm light, the bactericidal rate of PDA-Cur against *Escherichia coli* was as high as 100% at a concentration of 0.1 nM. In addition, PDA-Cur showed good biocompatibility by testing the cytotoxicity and hemolytic activity. In short, the PDA-Cur nanoparticles exhibit good photodynamic effects, photothermal function, and biocompatibility. The antibacterial activity of PDA-Cur was obviously stronger than that of free Cur in the presence of dual light. Therefore, the strategy of PDA-Cur nanocompounds shows that the combination of PDT and PTT can greatly enhance the antibacterial effect at low concentrations, providing a certain reference for the clinical application of PDT and PTT antibacterial in the future.

### Carbon dots nanoparticles

In view of the current difficulties faced by antibiotic treatment, researchers further developed the application of PDAT in antibacterial treatment. How to improve the antibacterial efficiency of traditional PS has become one of the focuses in the field of optoelectronic medicine. Yan (Yan et al., 2021b) obtained a dual-wavelength excited composite nanoparticle system CDs with synergistic photodynamic and photothermal bacteriostatic effects by using a hydrothermal method to synthesize fluorescent carbon dots (CDs) for loading curcumin (Cur). Under the combined irradiation of near-infrared and 405 nm light, CDs/Cur can generate a large amount of ROS and cause an increase in temperature, thereby triggering the synergistic antibacterial effect of PDT and PTT on Gram-positive and Gram-negative bacteria. The combined effect of CDs/Cur with PDT and PTT caused more serious damage to the cell membrane. In addition, CDs/Cur showed good biocompatibility. The CDs/Cur nanosystems constructed by utilizing CDs as photosensitizer delivery vehicles have promising application prospects, and also guide the design of future nanosystems for PS delivery.

### Graphene oxide nanomaterial

2D materials have been widely used in biomedical research due to their unique physical and chemical properties (Ouyang et al., 2022b). Graphene oxide (GO) possesses thermal, electrical, and antibacterial properties, and has broad application prospects in medicine (Romero et al., 2020). The generation of drug-resistant bacteria is considered a worldwide problem. Because of the PDT/PDD ability of graphene oxide, it is considered to be developed as a broad-spectrum selective antibacterial agent for the treatment of drug-resistant bacteria. María Paulina Romero et al. (2020) presents two different sizes of graphene oxide, including their preparation, characterization and optical properties determination. The *in vitro* light dose- and concentration-dependent PTT/PDT antibacterial effects of two graphene oxides were investigated by using Gram-negative *Escherichia coli* and Gram-positive *Staphylococcus aureus*. The results show that graphene oxide and nanographene oxide (NGO) are sensitive to O_2_ formation, can raise the temperature to 55°C–60°C, and are effective against Gram-positive *Staphylococcus aureus* and Gram-negative *Escherichia coli*. Sterilization plays a role in the synthesis of PTT/PDT. In this study, graphene oxide and NGO-based *S. aureus* and *Escherichia coli* were used at high concentrations of 43–47 J cm^−2^, and about 70 J cm^−2^ at low doses of graphene oxide and NGO. The presence of high concentrations of graphene oxide made the flora of *S. aureus* and *E. coli* more sensitive to the use of PDT/PTT, and the bactericidal effect of *S. aureus* and *E. coli* was similar to that of PDT. In neonatal skin fibroblast HDFs, graphene oxide did not significantly promote cell viability, but mild damage to HDF cells was observed in graphene oxide independent of concentration. The unique properties of graphene oxide and NGOs facilitate clinical treatment of broad-spectrum antibiotic antiseptics. The antibacterial effect of graphene oxide against gram-positive and gram-negative bacteria by PTT and PDT, using low light doses, led us to conclude that graphene oxide and NGO can be used for skin infections as this treatment is effective on human skin fibroblast effects are lower than antibacterial effects.

### Aggregation-induced fluorescence nanomaterial

Aggregation-induced fluorescence (AIE) molecules offer great advantages over aggregation-quenched fluorescence molecules (ACQs) in cellular imaging, image-guided PDT, and antimicrobial activity. However, the molecular design of AIEs for PDAT still faces significant challenges. Yang et al. (2020) reported a sequential strategy for the preparation of a series of AIE-active luminophores by changing the structure through a series of reliable reactions. After focusing on the main O_2_-generating factors, according to the longer emission wavelength of TPA-18, the energy gap between HOMO and LUMO is the lowest, which completely separates the orbitals of HOMO and LUMO. Distribution and typical AIE properties, TPA-18 was selected among all triphenylamine derivatives (TPA). Meanwhile, due to the structurally positive charge and bright emission color of TPA-18, the synthetic form of TPA-18 was found to be an excellent probe for capturing and imaging targeting typhoid. TPA-18 can efficiently generate ROS and can also provide effective guidance for image-guided PDT and antibacterial. Therefore, this study not only synthesized an AIE photoreceptor with controllable emission wavelength, but also proposed the concept of the design of multifunctional AIEgens.

Photosensitive substances with aggregation-induced fluorescence can generate ROS upon irradiation, showing great application prospects in the field of antibacterial materials. However, developing AlEgens with precisely regulated bacteriostatic activity remains a major challenge due to the limited molecular backbone. Guo et al. (2022) developed a series of AIEgens nanofibers by rationally designing peptides such as antimicrobial peptide (AMP) HHC36, ditryptophan, polyarginine, and polylysine as bacterial recognition ligands. These AlEgens exhibit precisely tunable antibacterial behaviors that can be modulated and inhibited by simply changing the modified peptides to recognize different bacteria. Mechanistic analysis indicated that this effect may be due to the synergistic antibacterial activity of ROS and peptides. It is worth noting that the optimized AIE-NFs NFs-K18 can inhibit methicillin-resistant *Staphylococcus aureus* (MRSA) and promote wound healing in living organisms under *in vitro* irradiation, and at the same time can effectively recognize and kill 4 A new clinical army. Synthetic design concept of AIE-NFs with precisely regulated antibacterial activity provides new ideas for targeting bacteria.

### Metal organic frameworks nanomaterial

Metal-Organic frameworks (MOFs) are regular crystal structures composed of metal nodes and organic ligands supports (Xu et al., 2021; Xue et al., 2021). People can arbitrarily design and assemble the structure, composition and properties of materials within a certain range according to their needs. Chen et al. (2020) uses only MOFs without any added antimicrobial components as non-antibiotic drugs for PDT treatment of chronic wound resistance (MDR) caused by multi-microbial infections. MOFs (PCN-224) were combined with Ti through a cation exchange strategy to obtain bimetallic PCN-224 (Zr/Ti), which was highly photoactive under visible light and could be antibacterial by generating ROS. Wound dressings were prepared by loading PCN-224 (Zr/Ti) NPs onto lactic acid-glycolic acid nanofibers with high biocompatibility and minimal cytotoxicity. Moreover, this work does not use other antibacterial agents, is simple and low-cost to prepare, and shows that MOFs for PADT have a strong ability to replace antibiotics.

### Silver nanomaterial

APDT has the disadvantage that it cannot be sustained, that is, the antibacterial effect will end soon after the end of the light exposure, so the researchers combined the material with the photodynamic therapy. Shitomi et al. (2020) prepared silver nanoclusters/rose bengal nanocomposite (AgNCs/RB) as a novel photosensitizer. The turbidity of *Streptococcus* mutans, Porphyromonas gingivalis and Aggregobacter actinomycetes was significantly decreased by short irradiation of AgNCs/RB with white light-emitting diodes (LED). Photoexcited AgNCs/RB reduced *Streptococcus* mutans colonization, disrupted cell membranes, and increased the number of dead cells. The antibacterial efficiency of photoexcited AgNCs/RB was higher than that of AgNCs or RB alone, suggesting that photoexcited AgNCs/RB had synergistic effects of ^1^0_2_ and Ag ions. Interestingly, the antimicrobial activity of AgNCs/RB against *Streptococcus* mutans remained unchanged even after LED irradiation was stopped, indicating a long-term antimicrobial effect of the released Ag ions.

### Nano-graphene quantum dots

Recent studies have shown that graphene quantum dots (GQDs) have photodynamic effects and can be used in APDT. Wang et al. (2022) prepared a composite system GQDs@hMSN(EM) by loading GQDs and erythromycin (EM) into hollow mesoporous silica nanoparticles (hMSN). Bacterial density experiments confirmed that GQDs@hMSN(EM) had photodynamic and drug-releasing antibacterial effects against *Escherichia coli* (*E. coli*) and *Staphylococcus aureus* (*S. aureus*). In animal models, the degree of wound healing of bacterial infection also confirmed that the GQDs@hMSN(EM) group had the best treatment effect, and the blood inflammatory factors were significantly reduced. GQDs@hMSN(EM) has good antimicrobial activity, sufficient drug loading capacity and controllable drug release ability, which provides a new opportunity for the GQDS-based nanoplatform to enhance antimicrobial efficacy and reduce drug resistance.

### Summary and outlook

Although PDAT shows great potential in antimicrobial therapy, there are still many studies to be done on the clinical application of PDAT due to the limitations of light penetration and the hypoxic environment at the site of infection. At the same time, PDAT is only suitable for local treatment, not systemic treatment, and the infection with the highest clinical mortality is systemic infection. PTT, like PDAT, is also suitable for local infections. In contrast to PTT, PDAT kills bacteria by generating strong oxidizing species called ROS. Due to the influence of ROS lifetime and propagation distance, compared with PTT, PDAT has less effect on the surrounding tissues. However, compared with photothermal agents, PS usually have a relatively short absorption wavelength, which is a disadvantage for the depth of treatment of photodynamic therapy. Nanomaterials have great potential to enhance PDAT, therefore, it is necessary to develop new nanomaterials for PS delivery. A large number of different types of nanomaterials are used in PDAT, however, the complexity of the building blocks that make up nanomaterials also prompts researchers to worry about their biosafety. Before the clinical transformation of nanomaterials, it is necessary to conduct more in-depth research on its biological safety (Ouyang et al., 2022c). Among all types of materials, organic and natural materials may be safer and more stable than inorganic materials, and are currently the first choice for nanomaterials development.

In summary, the development of a new combination of nanomaterials and PDAT should have several requirements, including nanomaterials biocompatibility, nanomaterials stability and nanomaterials high-efficiency targeting. Most importantly, the transformation of nanomaterials in infected tissue is safe and controllable for the enhancement of PDAT, which is also the advantage that ideal intelligent response materials should have.
